# Tetra­kis{2,4-bis­[(1-oxo-2-pyridyl)­sulfanyl­methyl]mesitylene} acetone hemisolvate 11.5-hydrate

**DOI:** 10.1107/S1600536808040233

**Published:** 2008-12-17

**Authors:** B. Ravindran Durai Nayagam, Samuel Robinson Jebas, P. Selvarathy Grace, Dieter Schollmeyer

**Affiliations:** a Department of Chemistry, Popes College, Sawyerpuram 628 251, Tamilnadu, India; b Department of Physics, Karunya University, Karunya Nagar, Coimbatore 641 114, India; c Institut für Organische Chemie, Universität Mainz, Duesbergweg 10-14, 55099 Mainz, Germany

## Abstract

In the crystal structure of the title compound, 4C_21_H_22_N_2_O_2_S_2_·0.5C_3_H_6_O·11.5H_2_O, there are four crystallographically independent mol­ecules (*A*, *B*, *C*, *D*) with similar geometries, 11 water mol­ecules and a solvent acetone mol­ecule which is disordered with a water mol­ecule with occupancy factors of 0.5:0.5. The dihedral angles formed by the mesitylene ring with the two pyridyl rings are 82.07 (3) and 78.39 (3)° in mol­ecule *A*, 86.20 (3) and 82.29 (3)° in mol­ecule *B*, 81.05 (3) and 76.0 (4)° in mol­ecule *C*, 86.0 (3) and 80.9 (3)° in moleule *D*. The two pyridyl rings form dihedral angles of 41.17 (4), 64.01 (3), 81.9 (3) and 82.25 (3)° in mol­ecules *A*, *B*, *C* and D, respectively. The crystal structure is stabilized by inter­molecular O—H⋯O hydrogen bonds and possible weak C—H⋯π inter­actions.  Some short intra­molecular S⋯O contacts are apparent [2.684 (4)–2.702 (4) Å].

## Related literature

For bond-length data, see: Allen *et al.* (1987 ▶). For biological activities of *N*-oxide derivatives, see: Bovin *et al.* (1992 ▶); Hartung *et al.* (1996 ▶); Katsuyuki *et al.* (1991 ▶); Leonard *et al.* (1955 ▶); Lobana & Bhatia (1989 ▶); Symons & West (1985 ▶). For related structures, see: Jebas *et al.* (2005 ▶); Ravindran Durai Nayagam *et al.* (2008 ▶).
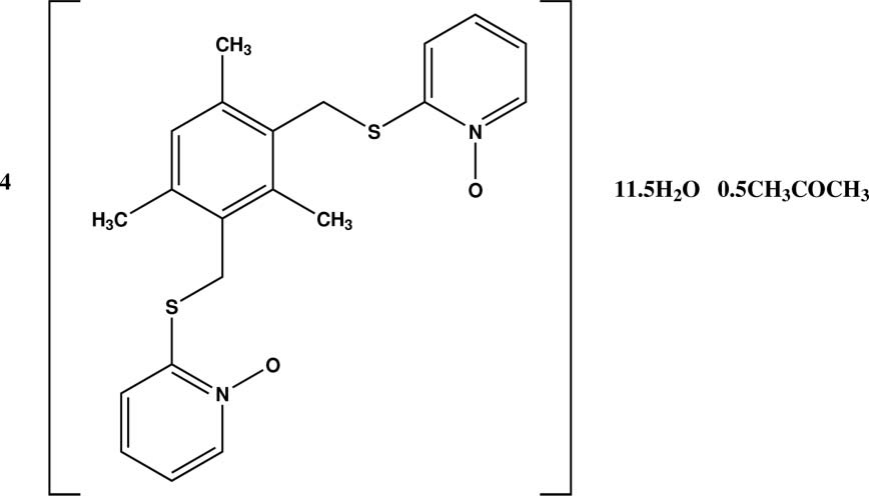


## Experimental

### 

#### Crystal data

4C_21_H_22_N_2_O_2_S_2_·0.5C_3_H_8_O_2_·11.5H_2_O
*M* *_r_* = 1830.41
Monoclinic, 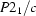

*a* = 19.1229 (12) Å
*b* = 28.3879 (19) Å
*c* = 17.3819 (11) Å
β = 102.808 (2)°
*V* = 9201.1 (10) Å^3^
*Z* = 4
Mo *K*α radiation
μ = 0.27 mm^−1^
*T* = 173 (2) K
0.45 × 0.15 × 0.10 mm

#### Data collection

Enraf–Nonius CAD-4 diffractometer
Absorption correction: none
126860 measured reflections
21877 independent reflections
10750 reflections with *I* > 2σ(*I*)
*R*_int_ = 0.117

#### Refinement

*R*[*F* ^2^ > 2σ(*F* ^2^)] = 0.094
*wR*(*F* ^2^) = 0.330
*S* = 1.02
21877 reflections
1129 parameters
98 restraints
H-atom parameters constrained
Δρ_max_ = 1.25 e Å^−3^
Δρ_min_ = −0.68 e Å^−3^

### 

Data collection: *CAD-4 EXPRESS* (Enraf–Nonius, 1994 ▶); cell refinement: *CAD-4 EXPRESS*; data reduction: *CORINC* (Dräger & Gattow, 1971 ▶); program(s) used to solve structure: *SHELXS97* (Sheldrick, 2008 ▶); program(s) used to refine structure: *SHELXL97* (Sheldrick, 2008 ▶); molecular graphics: *SHELXTL* (Sheldrick, 2008 ▶); software used to prepare material for publication: *SHELXTL* and *PLATON* (Spek, 2003 ▶).

## Supplementary Material

Crystal structure: contains datablocks global, I. DOI: 10.1107/S1600536808040233/bt2818sup1.cif

Structure factors: contains datablocks I. DOI: 10.1107/S1600536808040233/bt2818Isup2.hkl

Additional supplementary materials:  crystallographic information; 3D view; checkCIF report

## Figures and Tables

**Table 1:** Hydrogen-bond geometry (Å, °)

*D*—H⋯*A*	*D*—H	H⋯*A*	*D*⋯*A*	*D*—H⋯*A*
O1*W*—H1*W*⋯O18*A*^i^	0.84	1.98	2.800 (6)	167
O1*W*—H2*W*⋯O3*W*	0.84	2.13	2.832 (9)	141
O2*W*—H2*WA*⋯O4*W*	0.84	2.21	2.685 (11)	116
O2*W*—H2*WB*⋯O18*C*^ii^	0.84	1.87	2.666 (8)	157
O2*W*—H2*WB*⋯N17*C*^ii^	0.84	2.63	3.324 (8)	141
O3*W*—H3*WB*⋯O4*W*	0.85	2.00	2.849 (12)	179
O4*W*—H4*WA*⋯O9*W*	0.84	2.12	2.799 (17)	138
O4*W*—H4*WB*⋯O2*W*	0.84	2.07	2.685 (11)	129
O5*W*—H5*WA*⋯O27*C*^iii^	0.84	2.04	2.713 (14)	137
O5*W*—H5*WB*⋯O7*W*	0.84	2.15	2.817 (17)	137
O6*W*—H6*WB*⋯O5*W*	0.84	1.95	2.658 (16)	142
O7*W*—H7*WA*⋯O10*W*^iv^	0.84	2.28	2.74 (2)	115
O7*W*—H7*WB*⋯O5*W*	0.84	2.31	2.817 (17)	119
O8*W*—H8*WA*⋯O18*D*	0.84	2.08	2.819 (16)	147
O8*W*—H8*WA*⋯N17*D*	0.84	2.68	3.339 (17)	136
O8*W*—H8*WB*⋯O27*B*^v^	0.85	1.96	2.807 (15)	179
O9*W*—H9*WA*⋯O6*W*	0.84	2.42	3.028 (19)	130
O9*W*—H9*WB*⋯O11*W*	0.84	2.32	2.84 (3)	120
O10*W*—H10*W*⋯O11*W*^vi^	0.84	2.33	2.89 (3)	125
O10*W*—H10*X*⋯O7*W*^i^	0.84	2.10	2.74 (2)	133
O11*W*—H11*Y*⋯O9*W*	0.84	2.16	2.84 (3)	137
O12*W*—H12*W*⋯O27*D*	0.84	1.77	2.55 (2)	152
C14*B*—H14*B*⋯*Cg*1	0.95	2.93	3.548 (7)	124
C23*B*—H23*B*⋯*Cg*2	0.95	2.94	3.621 (7)	129
C8*A*—H8*A*3⋯*Cg*3	0.98	2.90	3.796 (6)	152

Symmetry codes: (i) 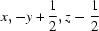; (ii) 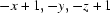; (iii) 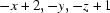; (iv) 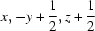; (v) 
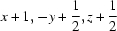
; (vi) 
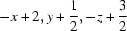
. *Cg*1, *Cg*2 and *Cg*3 are the centroids of the rings N17*A*/C12*A*–C16*A*, N26*A*/C21*A*–C25*A* and N26*B*/C21*B*–C25*B*, respectively.

**Fig. 1.**
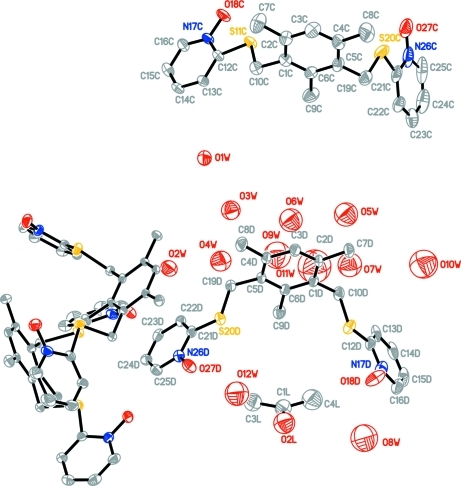
The molecular structure of the title compound, showing 50% probability displacement ellipsoids and the atom numbering scheme.

**Fig. 2.**
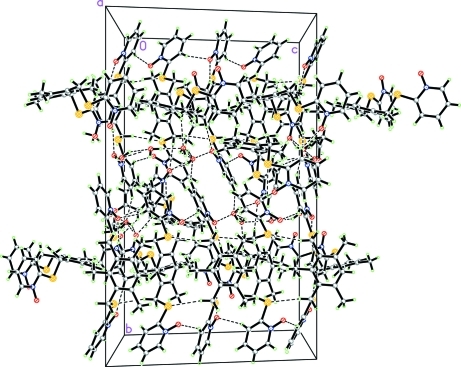
The crystal packing of the title compound, viewed down the a axis.

## supplementary crystallographic information

### Comment

N-oxides and their derivatives show a broad spectrum of biological activity such as antifungal, antimicrobial and antibacterial activities (Lobana & Bhatia, 1989; Symons & West, 1985). These compounds are also found to be involved in DNA strand scission under physiological conditions (Katsuyuki *et al.*, 1991; Bovin *et al.*, 1992). Pyridine N-oxides bearing a sulfur group in position two display significant antimicrobial activity (Leonard *et al.*, 1955). In view of the importance of N-oxides, we have previously reported the crystal structures of N-oxide derivatives (Jebas *et al.*, 2005; Ravindran Durai Nayagam *et al.*, 2008). As an extension of our work on N-oxide derivatives, we report here the crystal structure of the title compound.

The asymmetric unit of the title compound consists of four crystallographically independent molecules with similar geometries, 11 fully occupied water molecules and an acetone solvent molecule which is disordered with a water molecule with  occupancy factors of 0.5:0.5. The bond lengths and angles agree well with the N-oxide derivatives reported earlier (Jebas *et al.*, 2005) The N—O bond lengths are in good agreement with the mean value of 1.304 (15) Å reported in the literature for pyridine N-oxides (Allen *et al.*, 1987).

The dihedral angles formed by the mesitylene ring (C1A-C6A) with the two pyridyl rings (N17A/C12A-C16A; N26A/C21A-C25A) are 82.07 (3)° and 78.39 (3)° (molecule A); 86.20 (3)° and 82.29 (3)° (C1B-C6B) (N17B/C12B-C16B; N26B/C21B-C25B)(molecule B); 81.05 (3)° and 76.0 (4)° (C1C-C6C) (N17C/C12C-C16C; N26C/C21C-C25C)(molecule C); 86.0 (3)° and 80.9 (3)° (C1D-C6D) (N17D/C21D-C25D; N26D/C21D-C25D) (molecule D), respectively. The two pyridyl rings are inclined to each other forming a dihedral angles of 41.17 (4)° (N17A/C12A-C16A; N26A/C21A-C25A), 64.01 (3)° (N17B/C12B-C16B; N26B/C21B-C25B), 81.9 (3)° (N17A/C12C-C16C; N26C/C21C-C25C) and 82.25 (3)° (N17D/C21D-C25D; N26D/C21D-C25D) in molecules A,B,C & D respectively.

The crystal structure is stabilized by intramolecular, intermolecular O—H···O hydrogen bonds, C—H···π interactions together with intramolecular S···O = 2.684 (4) to 2.702 (4)Å short contacts (Table 1) (Fig 2).

### Experimental

A mixture of mono(bromomethyl)mesitylene (0.213 g, 1 mmol), 2,4-bis(bromomethyl)mesitylene (0.306 g, 1 mmol) and 1-hydroxypyridine-2-thione sodium salt (0.298,2 mmol) in water (30 ml) and methanol (30 ml) was heated at 333 K with stirring for 30 min. The compound formed was filtered off, and dried. The compound was dissolved in acetone and water (1:1 v/v) and allowed to undergo slow evaporation. Crystals were obtained after a week.

### Refinement

After checking their presence in the Fourier map, all H atoms were fixed on the calculated positions and allowed to ride on their parent atoms with C—H = 0.95 (aromatic), 0.99 (methylene) or 0.98 Å (methyl) and O—H = 0.84 Å with *U* set to 1.2U_eq_(C) or 1.5U_eq_(C_methyl_). The solvent acetone and a water molecule (O12W) are disordered and were refined with the fixed occupancy of 0.5:0.5.

### Crystal data

**Table:**

4C_21_H_22_N_2_O_2_S_2_·0.5C_3_H_8_O_2_·11.5H_2_O	*F*(000) = 3880
*M**_r_* = 1830.41	*D*_x_ = 1.321 Mg m^−^^3^
Monoclinic, *P*2_1_/*c*	Mo *K*α radiation, λ = 0.71069 Å
Hall symbol: -P 2ybc	Cell parameters from 8420 reflections
*a* = 19.1229 (12) Å	θ = 2.3–24.3°
*b* = 28.3879 (19) Å	µ = 0.27 mm^−^^1^
*c* = 17.3819 (11) Å	*T* = 173 K
β = 102.808 (2)°	Plate, colourless
*V* = 9201.1 (10) Å^3^	0.45 × 0.15 × 0.10 mm
*Z* = 4	

### Data collection

**Table:**

Enraf–Nonius CAD-4 diffractometer	10750 reflections with *I* > 2σ(*I*)
Radiation source: sealed tube	*R*_int_ = 0.117
graphite	θ_max_ = 27.9°, θ_min_ = 1.3°
CCD scan	*h* = −25→21
126860 measured reflections	*k* = −37→37
21877 independent reflections	*l* = −22→22

### Refinement

**Table:**

Refinement on *F*^2^	Primary atom site location: structure-invariant direct methods
Least-squares matrix: full	Secondary atom site location: difference Fourier map
*R*[*F*^2^ > 2σ(*F*^2^)] = 0.094	Hydrogen site location: inferred from neighbouring sites
*wR*(*F*^2^) = 0.330	H-atom parameters constrained
*S* = 1.02	*w* = 1/[σ^2^(*F*_o_^2^) + (0.1738*P*)^2^ + 12.516*P*] where *P* = (*F*_o_^2^ + 2*F*_c_^2^)/3
21877 reflections	(Δ/σ)_max_ < 0.001
1129 parameters	Δρ_max_ = 1.25 e Å^−^^3^
98 restraints	Δρ_min_ = −0.68 e Å^−^^3^

### Special details

**Table:**

Geometry. All e.s.d.'s (except the e.s.d. in the dihedral angle between two l.s. planes) are estimated using the full covariance matrix. The cell e.s.d.'s are taken into account individually in the estimation of e.s.d.'s in distances, angles and torsion angles; correlations between e.s.d.'s in cell parameters are only used when they are defined by crystal symmetry. An approximate (isotropic) treatment of cell e.s.d.'s is used for estimating e.s.d.'s involving l.s. planes.
Refinement. Refinement of *F*^2^ against ALL reflections. The weighted *R*-factor *wR* and goodness of fit *S* are based on *F*^2^, conventional *R*-factors *R* are based on *F*, with *F* set to zero for negative *F*^2^. The threshold expression of *F*^2^ > σ(*F*^2^) is used only for calculating *R*-factors(gt) *etc*. and is not relevant to the choice of reflections for refinement. *R*-factors based on *F*^2^ are statistically about twice as large as those based on *F*, and *R*- factors based on ALL data will be even larger.

### Fractional atomic coordinates and isotropic or equivalent isotropic displacement parameters (Å^2^)

**Table:**

	*x*	*y*	*z*	*U*_iso_*/*U*_eq_	Occ. (<1)
C1A	0.1828 (2)	0.29079 (17)	0.9645 (3)	0.0290 (10)	
C2A	0.1374 (2)	0.28957 (17)	0.8895 (3)	0.0301 (10)	
C3A	0.1158 (3)	0.24595 (18)	0.8563 (3)	0.0349 (11)	
H3A	0.0832	0.2451	0.8064	0.042*	
C4A	0.1395 (3)	0.20359 (19)	0.8924 (3)	0.0346 (11)	
C5A	0.1871 (2)	0.20534 (17)	0.9670 (3)	0.0304 (10)	
C6A	0.2091 (2)	0.24863 (17)	1.0029 (3)	0.0294 (10)	
C7A	0.1119 (3)	0.3334 (2)	0.8445 (3)	0.0445 (13)	
H7A1	0.0882	0.3252	0.7902	0.067*	
H7A2	0.1529	0.3540	0.8439	0.067*	
H7A3	0.0779	0.3498	0.8698	0.067*	
C8A	0.1143 (3)	0.1578 (2)	0.8523 (3)	0.0451 (13)	
H8A1	0.0815	0.1642	0.8015	0.068*	
H8A2	0.0893	0.1395	0.8858	0.068*	
H8A3	0.1557	0.1399	0.8436	0.068*	
C9A	0.2594 (3)	0.25019 (18)	1.0827 (3)	0.0350 (11)	
H9A1	0.2890	0.2787	1.0869	0.052*	
H9A2	0.2904	0.2223	1.0895	0.052*	
H9A3	0.2316	0.2506	1.1238	0.052*	
C10A	0.2043 (3)	0.33798 (17)	1.0033 (3)	0.0336 (11)	
H10A	0.2031	0.3367	1.0599	0.040*	
H10B	0.1709	0.3629	0.9777	0.040*	
S11A	0.29520 (7)	0.35087 (4)	0.99227 (8)	0.0360 (3)	
C12A	0.3052 (3)	0.40918 (18)	1.0254 (3)	0.0367 (11)	
C13A	0.2589 (3)	0.4348 (2)	1.0599 (4)	0.0474 (14)	
H13A	0.2173	0.4204	1.0710	0.057*	
C14A	0.2738 (4)	0.4818 (2)	1.0779 (4)	0.0634 (18)	
H14A	0.2428	0.4998	1.1021	0.076*	
C15A	0.3340 (4)	0.5024 (2)	1.0605 (4)	0.0590 (17)	
H15A	0.3434	0.5350	1.0701	0.071*	
C16A	0.3790 (3)	0.4756 (2)	1.0299 (4)	0.0522 (15)	
H16A	0.4212	0.4894	1.0195	0.063*	
N17A	0.3659 (2)	0.42974 (16)	1.0135 (3)	0.0407 (10)	
O18A	0.4105 (2)	0.40370 (15)	0.9827 (2)	0.0485 (10)	
C19A	0.2150 (3)	0.15975 (17)	1.0075 (3)	0.0352 (11)	
H19A	0.1843	0.1330	0.9840	0.042*	
H19B	0.2152	0.1614	1.0644	0.042*	
S20A	0.30564 (7)	0.15232 (4)	0.99311 (8)	0.0375 (3)	
C21A	0.3283 (3)	0.09730 (18)	1.0352 (3)	0.0393 (12)	
C22A	0.2876 (4)	0.06801 (19)	1.0723 (3)	0.0479 (14)	
H22A	0.2410	0.0774	1.0770	0.058*	
C23A	0.3156 (4)	0.0251 (2)	1.1023 (4)	0.0649 (19)	
H23A	0.2882	0.0046	1.1272	0.078*	
C24A	0.3830 (5)	0.0126 (3)	1.0956 (5)	0.074 (2)	
H24A	0.4026	−0.0167	1.1164	0.089*	
C25A	0.4226 (4)	0.0417 (2)	1.0594 (4)	0.0597 (17)	
H25A	0.4694	0.0325	1.0551	0.072*	
N26A	0.3956 (3)	0.08340 (16)	1.0296 (3)	0.0451 (11)	
O27A	0.4327 (2)	0.11184 (15)	0.9943 (2)	0.0521 (10)	
C1B	0.3794 (2)	0.29181 (16)	0.7636 (3)	0.0279 (10)	
C2B	0.4277 (2)	0.29248 (16)	0.7132 (3)	0.0286 (10)	
C3B	0.4518 (2)	0.25013 (17)	0.6889 (3)	0.0294 (10)	
H3B	0.4843	0.2508	0.6552	0.035*	
C4B	0.4297 (2)	0.20689 (16)	0.7127 (3)	0.0280 (10)	
C5B	0.3813 (2)	0.20607 (16)	0.7621 (3)	0.0279 (10)	
C6B	0.3555 (2)	0.24828 (16)	0.7871 (3)	0.0283 (9)	
C7B	0.4543 (3)	0.33786 (18)	0.6853 (3)	0.0405 (12)	
H7B1	0.4947	0.3497	0.7256	0.061*	
H7B2	0.4155	0.3612	0.6760	0.061*	
H7B3	0.4700	0.3323	0.6361	0.061*	
C8B	0.4584 (3)	0.16213 (18)	0.6839 (3)	0.0398 (12)	
H8B1	0.4935	0.1477	0.7272	0.060*	
H8B2	0.4815	0.1696	0.6404	0.060*	
H8B3	0.4188	0.1401	0.6655	0.060*	
C9B	0.3008 (3)	0.24724 (19)	0.8378 (3)	0.0382 (11)	
H9B1	0.2718	0.2186	0.8265	0.057*	
H9B2	0.2696	0.2749	0.8263	0.057*	
H9B3	0.3255	0.2475	0.8936	0.057*	
C10B	0.3535 (3)	0.33723 (17)	0.7913 (3)	0.0332 (11)	
H10C	0.3883	0.3628	0.7893	0.040*	
H10D	0.3479	0.3341	0.8463	0.040*	
S11B	0.26757 (7)	0.35051 (5)	0.72558 (8)	0.0377 (3)	
C12B	0.2455 (3)	0.40530 (18)	0.7586 (3)	0.0382 (12)	
C13B	0.2810 (3)	0.43125 (19)	0.8219 (3)	0.0406 (12)	
H13B	0.3242	0.4194	0.8542	0.049*	
C14B	0.2548 (4)	0.4742 (2)	0.8392 (4)	0.0572 (16)	
H14B	0.2797	0.4921	0.8831	0.069*	
C15B	0.1923 (4)	0.4911 (3)	0.7924 (5)	0.075 (2)	
H15B	0.1739	0.5208	0.8037	0.090*	
C16B	0.1566 (4)	0.4652 (2)	0.7299 (4)	0.067 (2)	
H16B	0.1132	0.4767	0.6978	0.080*	
N17B	0.1835 (3)	0.42248 (18)	0.7136 (3)	0.0499 (12)	
O18B	0.1489 (2)	0.39674 (17)	0.6537 (3)	0.0663 (13)	
C19B	0.3582 (3)	0.15936 (17)	0.7902 (3)	0.0329 (10)	
H19C	0.3529	0.1619	0.8454	0.039*	
H19D	0.3943	0.1348	0.7876	0.039*	
S20B	0.27253 (7)	0.14445 (5)	0.72502 (8)	0.0393 (3)	
C21B	0.2492 (3)	0.09375 (18)	0.7691 (3)	0.0349 (11)	
C22B	0.2879 (3)	0.07029 (17)	0.8353 (3)	0.0350 (11)	
H22B	0.3346	0.0810	0.8600	0.042*	
C23B	0.2592 (3)	0.0317 (2)	0.8653 (4)	0.0482 (14)	
H23B	0.2854	0.0158	0.9108	0.058*	
C24B	0.1902 (4)	0.0163 (2)	0.8272 (4)	0.0574 (16)	
H24B	0.1690	−0.0100	0.8471	0.069*	
C25B	0.1544 (3)	0.0392 (2)	0.7619 (4)	0.0559 (16)	
H25B	0.1081	0.0284	0.7358	0.067*	
N26B	0.1832 (2)	0.07725 (16)	0.7330 (3)	0.0408 (10)	
O27B	0.1471 (2)	0.09961 (17)	0.6701 (2)	0.0553 (11)	
C1C	0.7142 (4)	0.1111 (2)	0.2024 (4)	0.0526 (15)	
C2C	0.7057 (4)	0.1187 (3)	0.1208 (4)	0.0638 (18)	
C3C	0.7661 (5)	0.1270 (3)	0.0913 (5)	0.081 (2)	
H3C	0.7604	0.1319	0.0362	0.098*	
C4C	0.8339 (4)	0.1285 (3)	0.1386 (5)	0.069 (2)	
C5C	0.8430 (4)	0.1216 (2)	0.2216 (5)	0.0617 (18)	
C6C	0.7820 (4)	0.1122 (2)	0.2518 (4)	0.0532 (15)	
C7C	0.6323 (5)	0.1158 (4)	0.0647 (5)	0.108 (3)	
H7C1	0.6017	0.0940	0.0861	0.162*	
H7C2	0.6377	0.1045	0.0131	0.162*	
H7C3	0.6102	0.1472	0.0588	0.162*	
C8C	0.8968 (5)	0.1391 (4)	0.1023 (7)	0.110 (3)	
H8C1	0.8830	0.1340	0.0451	0.165*	
H8C2	0.9369	0.1183	0.1251	0.165*	
H8C3	0.9116	0.1720	0.1129	0.165*	
C9C	0.7924 (5)	0.1012 (3)	0.3397 (5)	0.086 (2)	
H9C1	0.7925	0.1306	0.3691	0.130*	
H9C2	0.8383	0.0849	0.3582	0.130*	
H9C3	0.7532	0.0810	0.3481	0.130*	
C10C	0.6482 (4)	0.0999 (2)	0.2332 (4)	0.0593 (17)	
H10E	0.6058	0.1163	0.2015	0.071*	
H10F	0.6549	0.1099	0.2889	0.071*	
S11C	0.63672 (9)	0.03638 (5)	0.22455 (10)	0.0514 (4)	
C12C	0.5644 (3)	0.02772 (19)	0.2683 (3)	0.0424 (13)	
C13C	0.5306 (3)	0.05998 (19)	0.3080 (3)	0.0436 (13)	
H13C	0.5463	0.0918	0.3125	0.052*	
C14C	0.4747 (3)	0.0463 (2)	0.3408 (3)	0.0489 (14)	
H14C	0.4517	0.0684	0.3680	0.059*	
C15C	0.4525 (4)	0.0002 (2)	0.3337 (4)	0.0596 (17)	
H15C	0.4135	−0.0098	0.3554	0.072*	
C16C	0.4867 (3)	−0.0316 (2)	0.2951 (4)	0.0544 (15)	
H16C	0.4714	−0.0635	0.2900	0.065*	
N17C	0.5416 (3)	−0.01772 (16)	0.2647 (3)	0.0475 (12)	
O18C	0.5769 (3)	−0.04903 (15)	0.2304 (3)	0.0674 (13)	
C19C	0.9155 (4)	0.1218 (3)	0.2752 (5)	0.078 (2)	
H19E	0.9116	0.1265	0.3305	0.093*	
H19F	0.9450	0.1476	0.2606	0.093*	
S20C	0.95587 (12)	0.06530 (8)	0.26411 (16)	0.0939 (8)	
C21C	1.0041 (4)	0.0522 (3)	0.3585 (6)	0.079 (2)	
C22C	1.0160 (4)	0.0793 (3)	0.4268 (5)	0.072 (2)	
H22C	0.9958	0.1099	0.4251	0.086*	
C23C	1.0572 (4)	0.0622 (4)	0.4983 (6)	0.089 (3)	
H23C	1.0650	0.0808	0.5448	0.107*	
C24C	1.0857 (5)	0.0186 (5)	0.4995 (9)	0.122 (4)	
H24C	1.1123	0.0063	0.5479	0.147*	
C25C	1.0767 (5)	−0.0092 (5)	0.4301 (12)	0.143 (6)	
H25C	1.0990	−0.0392	0.4314	0.171*	
N26C	1.0342 (4)	0.0084 (3)	0.3592 (7)	0.105 (3)	
O27C	1.0226 (3)	−0.0162 (2)	0.2924 (6)	0.138 (3)	
C1D	0.8467 (3)	0.2281 (2)	0.8069 (4)	0.0456 (13)	
C2D	0.8497 (3)	0.2383 (2)	0.7284 (4)	0.0502 (15)	
C3D	0.7856 (3)	0.2465 (2)	0.6742 (4)	0.0564 (16)	
H3D	0.7873	0.2542	0.6214	0.068*	
C4D	0.7188 (3)	0.2440 (2)	0.6941 (4)	0.0517 (15)	
C5D	0.7166 (3)	0.2327 (2)	0.7725 (4)	0.0447 (13)	
C6D	0.7806 (3)	0.22585 (19)	0.8290 (4)	0.0439 (13)	
C7D	0.9192 (4)	0.2394 (3)	0.7015 (5)	0.070 (2)	
H7D1	0.9093	0.2446	0.6444	0.105*	
H7D2	0.9443	0.2094	0.7142	0.105*	
H7D3	0.9492	0.2651	0.7285	0.105*	
C8D	0.6514 (4)	0.2517 (3)	0.6306 (4)	0.075 (2)	
H8D1	0.6645	0.2629	0.5823	0.112*	
H8D2	0.6211	0.2753	0.6486	0.112*	
H8D3	0.6250	0.2220	0.6198	0.112*	
C9D	0.7774 (4)	0.2164 (3)	0.9135 (4)	0.0593 (17)	
H9D1	0.7398	0.2359	0.9276	0.089*	
H9D2	0.8238	0.2243	0.9482	0.089*	
H9D3	0.7667	0.1831	0.9197	0.089*	
C10D	0.9152 (3)	0.2189 (2)	0.8675 (4)	0.0554 (16)	
H10G	0.9526	0.2065	0.8416	0.066*	
H10H	0.9067	0.1953	0.9064	0.066*	
S11D	0.94381 (9)	0.27323 (6)	0.91574 (11)	0.0601 (5)	
C12D	1.0052 (3)	0.2542 (3)	1.0004 (4)	0.0633 (19)	
C13D	1.0248 (3)	0.2096 (3)	1.0250 (4)	0.066 (2)	
H13D	1.0057	0.1834	0.9932	0.079*	
C14D	1.0731 (4)	0.2025 (4)	1.0972 (5)	0.079 (3)	
H14D	1.0852	0.1713	1.1153	0.095*	
C15D	1.1029 (4)	0.2398 (5)	1.1420 (5)	0.105 (4)	
H15D	1.1373	0.2349	1.1899	0.126*	
C16D	1.0824 (4)	0.2844 (5)	1.1168 (5)	0.094 (3)	
H16D	1.1012	0.3108	1.1482	0.113*	
N17D	1.0347 (3)	0.2908 (3)	1.0462 (4)	0.080 (2)	
O18D	1.0153 (4)	0.3326 (3)	1.0222 (4)	0.109 (2)	
C19D	0.6460 (3)	0.2274 (2)	0.7959 (4)	0.0474 (14)	
H19G	0.6500	0.2033	0.8378	0.057*	
H19H	0.6087	0.2171	0.7499	0.057*	
S20D	0.62144 (8)	0.28440 (5)	0.83182 (9)	0.0477 (4)	
C21D	0.5598 (3)	0.2675 (2)	0.8869 (3)	0.0410 (12)	
C22D	0.5383 (3)	0.2225 (2)	0.9038 (3)	0.0429 (13)	
H22D	0.5573	0.1957	0.8826	0.051*	
C23D	0.4897 (3)	0.2165 (2)	0.9509 (3)	0.0482 (14)	
H23D	0.4754	0.1858	0.9630	0.058*	
C24D	0.4624 (3)	0.2559 (2)	0.9801 (3)	0.0478 (14)	
H24D	0.4293	0.2522	1.0131	0.057*	
C25D	0.4823 (3)	0.3002 (2)	0.9625 (3)	0.0477 (14)	
H25D	0.4619	0.3271	0.9816	0.057*	
N26D	0.5311 (2)	0.30539 (16)	0.9175 (3)	0.0435 (11)	
O27D	0.5517 (3)	0.34769 (15)	0.9017 (3)	0.0615 (12)	
O1W	0.5593 (3)	0.08658 (18)	0.5339 (3)	0.0748 (14)	
H1W	0.5149	0.0851	0.5153	0.112*	
H2W	0.5674	0.1045	0.5733	0.112*	
O2W	0.5159 (3)	0.0854 (2)	0.8926 (4)	0.1020 (19)	
H2WA	0.5242	0.1094	0.8677	0.153*	
H2WB	0.4835	0.0693	0.8639	0.153*	
O3W	0.6392 (4)	0.1123 (3)	0.6854 (5)	0.130 (3)	
H3WA	0.6650	0.0895	0.6834	0.195*	
H3WB	0.6369	0.1131	0.7337	0.195*	
O4W	0.6339 (5)	0.1156 (3)	0.8479 (5)	0.149 (3)	
H4WA	0.6634	0.0934	0.8588	0.224*	
H4WB	0.5926	0.1046	0.8308	0.224*	
O5W	0.9258 (6)	0.0976 (4)	0.7553 (7)	0.200 (4)	
H5WA	0.9527	0.0740	0.7657	0.300*	
H5WB	0.9132	0.1065	0.7963	0.300*	
O6W	0.7882 (6)	0.1220 (4)	0.7263 (7)	0.193 (4)	
H6WA	0.7757	0.1076	0.6832	0.289*	
H6WB	0.8252	0.1093	0.7536	0.289*	
O7W	0.9543 (6)	0.1005 (4)	0.9214 (7)	0.205 (5)	
H7WA	0.9914	0.0874	0.9133	0.307*	
H7WB	0.9187	0.0924	0.8861	0.307*	
O8W	1.0027 (8)	0.3781 (5)	1.1626 (9)	0.265 (7)	
H8WA	0.9882	0.3636	1.1199	0.397*	
H8WB	1.0465	0.3847	1.1653	0.397*	
O9W	0.7761 (8)	0.0842 (5)	0.8857 (9)	0.261 (7)	
H9WA	0.7846	0.0757	0.8424	0.391*	
H9WB	0.8147	0.0916	0.9170	0.391*	
O10W	1.0676 (9)	0.4403 (6)	0.5238 (10)	0.288 (7)	
H10W	1.0605	0.4680	0.5372	0.431*	
H10X	1.0298	0.4246	0.5196	0.431*	
O11W	0.8815 (12)	0.0344 (8)	0.9965 (15)	0.400 (12)	
H11W	0.8674	0.0158	1.0275	0.600*	
H11Y	0.8516	0.0347	0.9530	0.600*	
C1L	0.7927 (8)	0.3770 (7)	1.0364 (12)	0.099 (6)	0.50
O2L	0.7923 (10)	0.4213 (7)	1.0331 (12)	0.162 (7)	0.50
C3L	0.7200 (9)	0.3567 (8)	1.0329 (14)	0.112 (7)	0.50
H3L1	0.7083	0.3534	1.0848	0.167*	0.50
H3L2	0.7161	0.3259	1.0068	0.167*	0.50
H3L3	0.6865	0.3788	1.0007	0.167*	0.50
C4L	0.8648 (11)	0.3539 (9)	1.0470 (17)	0.151 (10)	0.50
H4L1	0.8814	0.3583	0.9980	0.227*	0.50
H4L2	0.8607	0.3202	1.0570	0.227*	0.50
H4L3	0.8992	0.3683	1.0910	0.227*	0.50
O12W	0.6717 (13)	0.3857 (8)	0.9632 (14)	0.203 (9)	0.50
H12W	0.6333	0.3791	0.9309	0.305*	0.50
H12X	0.6820	0.3639	0.9964	0.305*	0.50

### Atomic displacement parameters (Å^2^)

**Table:**

	*U*^11^	*U*^22^	*U*^33^	*U*^12^	*U*^13^	*U*^23^
C1A	0.021 (2)	0.039 (3)	0.028 (2)	0.0038 (19)	0.0085 (18)	0.0005 (19)
C2A	0.021 (2)	0.042 (3)	0.029 (2)	0.0032 (19)	0.0091 (19)	0.004 (2)
C3A	0.025 (2)	0.054 (3)	0.026 (2)	0.001 (2)	0.0071 (19)	0.000 (2)
C4A	0.027 (2)	0.049 (3)	0.030 (3)	−0.004 (2)	0.011 (2)	−0.006 (2)
C5A	0.030 (2)	0.034 (2)	0.029 (2)	−0.0029 (19)	0.010 (2)	0.0000 (19)
C6A	0.024 (2)	0.041 (3)	0.025 (2)	0.000 (2)	0.0083 (18)	−0.002 (2)
C7A	0.040 (3)	0.055 (3)	0.037 (3)	0.011 (3)	0.006 (2)	0.010 (2)
C8A	0.043 (3)	0.053 (3)	0.037 (3)	−0.003 (3)	0.004 (2)	−0.012 (2)
C9A	0.032 (2)	0.042 (3)	0.029 (2)	0.002 (2)	0.002 (2)	0.002 (2)
C10A	0.032 (2)	0.037 (3)	0.033 (3)	0.000 (2)	0.012 (2)	−0.002 (2)
S11A	0.0328 (6)	0.0377 (7)	0.0411 (7)	−0.0024 (5)	0.0160 (5)	−0.0024 (5)
C12A	0.039 (3)	0.042 (3)	0.029 (3)	−0.009 (2)	0.006 (2)	−0.001 (2)
C13A	0.043 (3)	0.049 (3)	0.053 (3)	−0.008 (3)	0.015 (3)	−0.014 (3)
C14A	0.062 (4)	0.060 (4)	0.071 (5)	−0.003 (3)	0.022 (4)	−0.025 (3)
C15A	0.068 (4)	0.047 (3)	0.060 (4)	−0.018 (3)	0.008 (3)	−0.011 (3)
C16A	0.057 (4)	0.054 (4)	0.045 (3)	−0.021 (3)	0.011 (3)	−0.007 (3)
N17A	0.039 (2)	0.052 (3)	0.032 (2)	−0.012 (2)	0.0103 (19)	0.000 (2)
O18A	0.041 (2)	0.063 (3)	0.046 (2)	−0.0110 (19)	0.0195 (18)	−0.0042 (19)
C19A	0.036 (3)	0.037 (3)	0.031 (3)	−0.004 (2)	0.006 (2)	0.003 (2)
S20A	0.0434 (7)	0.0348 (6)	0.0373 (7)	0.0060 (5)	0.0153 (6)	0.0058 (5)
C21A	0.052 (3)	0.036 (3)	0.029 (3)	0.008 (2)	0.006 (2)	−0.002 (2)
C22A	0.066 (4)	0.036 (3)	0.041 (3)	0.000 (3)	0.011 (3)	0.004 (2)
C23A	0.089 (5)	0.042 (3)	0.061 (4)	0.000 (3)	0.010 (4)	0.015 (3)
C24A	0.097 (6)	0.050 (4)	0.070 (5)	0.023 (4)	0.010 (4)	0.018 (4)
C25A	0.069 (4)	0.046 (3)	0.059 (4)	0.020 (3)	0.002 (3)	0.000 (3)
N26A	0.055 (3)	0.041 (2)	0.039 (3)	0.012 (2)	0.009 (2)	−0.001 (2)
O27A	0.054 (2)	0.054 (2)	0.054 (2)	0.011 (2)	0.024 (2)	0.000 (2)
C1B	0.024 (2)	0.035 (2)	0.022 (2)	0.0044 (18)	0.0010 (18)	−0.0006 (19)
C2B	0.025 (2)	0.036 (2)	0.024 (2)	−0.0039 (19)	0.0051 (18)	0.0021 (19)
C3B	0.023 (2)	0.039 (2)	0.026 (2)	0.0013 (19)	0.0052 (18)	−0.002 (2)
C4B	0.026 (2)	0.036 (2)	0.020 (2)	0.0039 (19)	0.0025 (18)	−0.0018 (18)
C5B	0.023 (2)	0.033 (2)	0.026 (2)	−0.0003 (18)	0.0037 (18)	0.0045 (19)
C6B	0.025 (2)	0.038 (2)	0.022 (2)	0.0025 (19)	0.0074 (17)	0.0039 (19)
C7B	0.041 (3)	0.041 (3)	0.043 (3)	−0.006 (2)	0.014 (2)	0.003 (2)
C8B	0.039 (3)	0.042 (3)	0.041 (3)	0.008 (2)	0.014 (2)	−0.006 (2)
C9B	0.037 (3)	0.047 (3)	0.035 (3)	0.003 (2)	0.017 (2)	0.006 (2)
C10B	0.030 (2)	0.038 (3)	0.031 (3)	0.001 (2)	0.006 (2)	−0.002 (2)
S11B	0.0388 (7)	0.0389 (7)	0.0331 (7)	0.0101 (5)	0.0034 (5)	−0.0037 (5)
C12B	0.038 (3)	0.039 (3)	0.040 (3)	0.012 (2)	0.012 (2)	0.005 (2)
C13B	0.044 (3)	0.043 (3)	0.034 (3)	0.006 (2)	0.009 (2)	−0.001 (2)
C14B	0.062 (4)	0.051 (4)	0.055 (4)	0.012 (3)	0.005 (3)	−0.012 (3)
C15B	0.081 (5)	0.060 (4)	0.081 (5)	0.035 (4)	0.010 (4)	−0.015 (4)
C16B	0.057 (4)	0.067 (4)	0.070 (5)	0.029 (3)	−0.001 (3)	−0.013 (4)
N17B	0.052 (3)	0.053 (3)	0.043 (3)	0.017 (2)	0.007 (2)	−0.006 (2)
O18B	0.051 (3)	0.073 (3)	0.065 (3)	0.020 (2)	−0.009 (2)	−0.019 (2)
C19B	0.030 (2)	0.033 (2)	0.035 (3)	−0.0011 (19)	0.006 (2)	0.000 (2)
S20B	0.0338 (7)	0.0471 (7)	0.0343 (7)	−0.0053 (6)	0.0013 (5)	0.0100 (6)
C21B	0.035 (3)	0.037 (3)	0.035 (3)	−0.001 (2)	0.014 (2)	−0.003 (2)
C22B	0.034 (3)	0.033 (3)	0.039 (3)	0.000 (2)	0.011 (2)	−0.003 (2)
C23B	0.062 (4)	0.041 (3)	0.044 (3)	−0.005 (3)	0.016 (3)	0.000 (2)
C24B	0.065 (4)	0.050 (4)	0.059 (4)	−0.020 (3)	0.018 (3)	−0.001 (3)
C25B	0.046 (3)	0.062 (4)	0.059 (4)	−0.023 (3)	0.011 (3)	−0.006 (3)
N26B	0.033 (2)	0.048 (3)	0.039 (2)	−0.0086 (19)	0.0043 (19)	−0.003 (2)
O27B	0.035 (2)	0.084 (3)	0.044 (2)	−0.004 (2)	0.0017 (18)	0.008 (2)
C1C	0.064 (4)	0.037 (3)	0.060 (4)	0.000 (3)	0.022 (3)	0.009 (3)
C2C	0.065 (4)	0.066 (4)	0.059 (4)	−0.003 (3)	0.012 (3)	0.017 (3)
C3C	0.096 (6)	0.089 (6)	0.060 (5)	−0.024 (5)	0.017 (4)	0.018 (4)
C4C	0.068 (5)	0.067 (4)	0.081 (5)	−0.014 (4)	0.035 (4)	0.011 (4)
C5C	0.054 (4)	0.048 (4)	0.082 (5)	−0.015 (3)	0.012 (4)	0.002 (3)
C6C	0.060 (4)	0.043 (3)	0.054 (4)	−0.006 (3)	0.009 (3)	0.004 (3)
C7C	0.089 (6)	0.147 (9)	0.075 (6)	−0.019 (6)	−0.011 (5)	0.040 (6)
C8C	0.102 (7)	0.113 (8)	0.130 (9)	−0.033 (6)	0.060 (7)	0.017 (6)
C9C	0.098 (6)	0.096 (6)	0.060 (5)	−0.019 (5)	0.007 (4)	0.004 (4)
C10C	0.063 (4)	0.044 (3)	0.074 (5)	0.002 (3)	0.022 (4)	0.007 (3)
S11C	0.0588 (9)	0.0413 (8)	0.0623 (10)	0.0041 (7)	0.0314 (8)	−0.0012 (7)
C12C	0.047 (3)	0.042 (3)	0.043 (3)	0.010 (2)	0.019 (3)	0.004 (2)
C13C	0.049 (3)	0.035 (3)	0.047 (3)	0.011 (2)	0.012 (3)	0.002 (2)
C14C	0.056 (4)	0.053 (3)	0.042 (3)	0.019 (3)	0.021 (3)	0.002 (3)
C15C	0.057 (4)	0.066 (4)	0.064 (4)	0.011 (3)	0.030 (3)	0.009 (3)
C16C	0.053 (4)	0.049 (3)	0.067 (4)	0.002 (3)	0.026 (3)	−0.001 (3)
N17C	0.052 (3)	0.040 (2)	0.057 (3)	0.006 (2)	0.025 (2)	−0.006 (2)
O18C	0.076 (3)	0.048 (2)	0.094 (4)	0.001 (2)	0.052 (3)	−0.019 (2)
C19C	0.065 (5)	0.060 (4)	0.106 (6)	−0.019 (4)	0.017 (4)	−0.015 (4)
S20C	0.0694 (13)	0.0738 (13)	0.1220 (19)	−0.0054 (10)	−0.0143 (13)	−0.0388 (13)
C21C	0.041 (4)	0.053 (4)	0.138 (8)	−0.018 (3)	0.010 (4)	−0.006 (4)
C22C	0.057 (4)	0.065 (4)	0.092 (6)	−0.021 (3)	0.013 (4)	0.004 (4)
C23C	0.060 (5)	0.099 (7)	0.114 (8)	−0.005 (5)	0.029 (5)	0.029 (6)
C24C	0.063 (6)	0.129 (11)	0.169 (13)	−0.020 (7)	0.013 (7)	0.056 (9)
C25C	0.046 (5)	0.099 (9)	0.28 (2)	−0.002 (5)	0.034 (9)	0.057 (11)
N26C	0.042 (4)	0.066 (5)	0.206 (10)	−0.013 (3)	0.025 (5)	−0.005 (6)
O27C	0.060 (4)	0.086 (4)	0.259 (10)	−0.014 (3)	0.020 (5)	−0.074 (6)
C1D	0.035 (3)	0.044 (3)	0.054 (4)	0.002 (2)	0.003 (3)	−0.007 (3)
C2D	0.027 (3)	0.067 (4)	0.057 (4)	−0.002 (3)	0.010 (3)	−0.005 (3)
C3D	0.048 (4)	0.078 (5)	0.044 (3)	−0.005 (3)	0.012 (3)	−0.001 (3)
C4D	0.033 (3)	0.071 (4)	0.049 (3)	−0.001 (3)	0.005 (3)	−0.005 (3)
C5D	0.032 (3)	0.049 (3)	0.053 (3)	0.000 (2)	0.010 (3)	−0.007 (3)
C6D	0.036 (3)	0.041 (3)	0.054 (3)	−0.002 (2)	0.007 (3)	−0.002 (3)
C7D	0.042 (4)	0.104 (6)	0.069 (5)	−0.009 (4)	0.022 (3)	−0.005 (4)
C8D	0.047 (4)	0.118 (7)	0.053 (4)	0.003 (4)	−0.002 (3)	0.001 (4)
C9D	0.054 (4)	0.071 (4)	0.050 (4)	0.000 (3)	0.005 (3)	0.010 (3)
C10D	0.040 (3)	0.055 (4)	0.068 (4)	0.009 (3)	0.004 (3)	0.005 (3)
S11D	0.0495 (9)	0.0620 (10)	0.0604 (10)	−0.0073 (8)	−0.0058 (8)	0.0062 (8)
C12D	0.038 (3)	0.103 (6)	0.051 (4)	−0.018 (4)	0.013 (3)	0.002 (4)
C13D	0.038 (3)	0.105 (6)	0.061 (4)	0.015 (3)	0.024 (3)	0.030 (4)
C14D	0.045 (4)	0.140 (8)	0.058 (5)	0.026 (5)	0.023 (4)	0.038 (5)
C15D	0.041 (4)	0.230 (14)	0.046 (5)	−0.005 (6)	0.015 (4)	0.025 (7)
C16D	0.056 (5)	0.182 (11)	0.046 (4)	−0.035 (6)	0.013 (4)	−0.011 (6)
N17D	0.058 (4)	0.121 (6)	0.059 (4)	−0.026 (4)	0.008 (3)	0.012 (4)
O18D	0.129 (6)	0.101 (5)	0.078 (4)	−0.041 (4)	−0.016 (4)	−0.008 (4)
C19D	0.039 (3)	0.049 (3)	0.055 (4)	0.000 (3)	0.011 (3)	−0.005 (3)
S20D	0.0416 (8)	0.0481 (8)	0.0562 (9)	0.0020 (6)	0.0168 (7)	0.0058 (7)
C21D	0.031 (3)	0.054 (3)	0.035 (3)	0.005 (2)	0.002 (2)	0.007 (2)
C22D	0.035 (3)	0.049 (3)	0.042 (3)	0.003 (2)	0.003 (2)	0.000 (2)
C23D	0.041 (3)	0.060 (4)	0.040 (3)	−0.014 (3)	0.002 (3)	0.009 (3)
C24D	0.045 (3)	0.069 (4)	0.028 (3)	0.000 (3)	0.005 (2)	0.000 (3)
C25D	0.048 (3)	0.058 (4)	0.036 (3)	0.013 (3)	0.008 (3)	0.003 (3)
N26D	0.043 (3)	0.045 (3)	0.041 (3)	0.004 (2)	0.007 (2)	0.009 (2)
O27D	0.075 (3)	0.048 (2)	0.071 (3)	0.009 (2)	0.036 (3)	0.008 (2)
O1W	0.069 (2)	0.079 (2)	0.076 (2)	−0.0017 (16)	0.0165 (16)	0.0021 (16)
O2W	0.098 (2)	0.109 (2)	0.098 (2)	0.0162 (18)	0.0213 (18)	−0.0101 (18)
O3W	0.130 (3)	0.133 (3)	0.128 (3)	−0.0046 (19)	0.0297 (19)	−0.0010 (19)
O4W	0.156 (3)	0.146 (3)	0.146 (3)	−0.0083 (19)	0.034 (2)	0.0006 (19)
O5W	0.199 (5)	0.199 (5)	0.201 (5)	−0.001 (2)	0.043 (2)	−0.001 (2)
O6W	0.191 (5)	0.191 (5)	0.195 (5)	0.001 (2)	0.041 (2)	−0.001 (2)
O7W	0.206 (5)	0.204 (5)	0.205 (5)	−0.003 (2)	0.046 (2)	0.000 (2)
O8W	0.265 (7)	0.265 (7)	0.264 (7)	−0.002 (2)	0.060 (2)	0.000 (2)
O9W	0.263 (7)	0.259 (7)	0.261 (7)	−0.001 (2)	0.059 (2)	−0.002 (2)
O10W	0.289 (8)	0.286 (8)	0.286 (8)	0.001 (2)	0.060 (3)	0.001 (2)
O11W	0.400 (12)	0.400 (12)	0.400 (12)	−0.002 (2)	0.089 (3)	0.000 (2)
O12W	0.204 (9)	0.203 (9)	0.203 (9)	−0.001 (2)	0.046 (3)	0.000 (2)
C1L	0.099 (6)	0.099 (6)	0.099 (6)	−0.001 (2)	0.021 (2)	0.001 (2)
O2L	0.162 (7)	0.161 (7)	0.162 (7)	0.001 (2)	0.034 (2)	0.000 (2)
C3L	0.111 (7)	0.112 (7)	0.112 (7)	0.000 (2)	0.024 (2)	0.000 (2)
C4L	0.152 (10)	0.151 (10)	0.151 (10)	0.002 (2)	0.034 (3)	0.001 (2)

### Geometric parameters (Å, °)

**Table:**

C1A—C2A	1.396 (6)	C8C—H8C3	0.9800
C1A—C6A	1.408 (7)	C9C—H9C1	0.9800
C1A—C10A	1.516 (7)	C9C—H9C2	0.9800
C2A—C3A	1.390 (7)	C9C—H9C3	0.9800
C2A—C7A	1.494 (7)	C10C—S11C	1.820 (6)
C3A—C4A	1.385 (7)	C10C—H10E	0.9900
C3A—H3A	0.9500	C10C—H10F	0.9900
C4A—C5A	1.410 (7)	S11C—C12C	1.736 (6)
C4A—C8A	1.502 (7)	C12C—N17C	1.359 (7)
C5A—C6A	1.400 (7)	C12C—C13C	1.389 (7)
C5A—C19A	1.512 (7)	C13C—C14C	1.375 (8)
C6A—C9A	1.504 (7)	C13C—H13C	0.9500
C7A—H7A1	0.9800	C14C—C15C	1.373 (9)
C7A—H7A2	0.9800	C14C—H14C	0.9500
C7A—H7A3	0.9800	C15C—C16C	1.374 (9)
C8A—H8A1	0.9800	C15C—H15C	0.9500
C8A—H8A2	0.9800	C16C—N17C	1.335 (7)
C8A—H8A3	0.9800	C16C—H16C	0.9500
C9A—H9A1	0.9800	N17C—O18C	1.334 (6)
C9A—H9A2	0.9800	C19C—S20C	1.809 (8)
C9A—H9A3	0.9800	C19C—H19E	0.9900
C10A—S11A	1.827 (5)	C19C—H19F	0.9900
C10A—H10A	0.9900	S20C—C21C	1.734 (10)
C10A—H10B	0.9900	C21C—N26C	1.370 (10)
S11A—C12A	1.749 (5)	C21C—C22C	1.390 (11)
C12A—N17A	1.356 (6)	C22C—C23C	1.402 (12)
C12A—C13A	1.382 (8)	C22C—H22C	0.9500
C13A—C14A	1.386 (9)	C23C—C24C	1.348 (16)
C13A—H13A	0.9500	C23C—H23C	0.9500
C14A—C15A	1.383 (9)	C24C—C25C	1.421 (19)
C14A—H14A	0.9500	C24C—H24C	0.9500
C15A—C16A	1.345 (9)	C25C—N26C	1.408 (17)
C15A—H15A	0.9500	C25C—H25C	0.9500
C16A—N17A	1.343 (7)	N26C—O27C	1.330 (12)
C16A—H16A	0.9500	C1D—C6D	1.403 (8)
N17A—O18A	1.328 (6)	C1D—C2D	1.407 (9)
C19A—S20A	1.818 (5)	C1D—C10D	1.511 (8)
C19A—H19A	0.9900	C2D—C3D	1.391 (9)
C19A—H19B	0.9900	C2D—C7D	1.504 (9)
S20A—C21A	1.738 (5)	C3D—C4D	1.398 (9)
C21A—N26A	1.370 (7)	C3D—H3D	0.9500
C21A—C22A	1.392 (8)	C4D—C5D	1.408 (9)
C22A—C23A	1.385 (8)	C4D—C8D	1.516 (9)
C22A—H22A	0.9500	C5D—C6D	1.402 (8)
C23A—C24A	1.367 (10)	C5D—C19D	1.502 (8)
C23A—H23A	0.9500	C6D—C9D	1.507 (9)
C24A—C25A	1.365 (10)	C7D—H7D1	0.9800
C24A—H24A	0.9500	C7D—H7D2	0.9800
C25A—N26A	1.348 (7)	C7D—H7D3	0.9800
C25A—H25A	0.9500	C8D—H8D1	0.9800
N26A—O27A	1.313 (6)	C8D—H8D2	0.9800
C1B—C2B	1.406 (6)	C8D—H8D3	0.9800
C1B—C6B	1.409 (7)	C9D—H9D1	0.9800
C1B—C10B	1.498 (7)	C9D—H9D2	0.9800
C2B—C3B	1.386 (7)	C9D—H9D3	0.9800
C2B—C7B	1.505 (7)	C10D—S11D	1.783 (7)
C3B—C4B	1.390 (7)	C10D—H10G	0.9900
C3B—H3B	0.9500	C10D—H10H	0.9900
C4B—C5B	1.395 (6)	S11D—C12D	1.752 (7)
C4B—C8B	1.513 (7)	C12D—N17D	1.355 (10)
C5B—C6B	1.401 (7)	C12D—C13D	1.362 (10)
C5B—C19B	1.512 (6)	C13D—C14D	1.398 (10)
C6B—C9B	1.511 (7)	C13D—H13D	0.9500
C7B—H7B1	0.9800	C14D—C15D	1.361 (14)
C7B—H7B2	0.9800	C14D—H14D	0.9500
C7B—H7B3	0.9800	C15D—C16D	1.368 (15)
C8B—H8B1	0.9800	C15D—H15D	0.9500
C8B—H8B2	0.9800	C16D—N17D	1.369 (11)
C8B—H8B3	0.9800	C16D—H16D	0.9500
C9B—H9B1	0.9800	N17D—O18D	1.284 (9)
C9B—H9B2	0.9800	C19D—S20D	1.833 (6)
C9B—H9B3	0.9800	C19D—H19G	0.9900
C10B—S11B	1.821 (5)	C19D—H19H	0.9900
C10B—H10C	0.9900	S20D—C21D	1.744 (6)
C10B—H10D	0.9900	C21D—N26D	1.368 (7)
S11B—C12B	1.742 (5)	C21D—C22D	1.392 (8)
C12B—N17B	1.357 (7)	C22D—C23D	1.378 (8)
C12B—C13B	1.373 (8)	C22D—H22D	0.9500
C13B—C14B	1.378 (8)	C23D—C24D	1.377 (9)
C13B—H13B	0.9500	C23D—H23D	0.9500
C14B—C15B	1.374 (9)	C24D—C25D	1.369 (8)
C14B—H14B	0.9500	C24D—H24D	0.9500
C15B—C16B	1.363 (10)	C25D—N26D	1.351 (7)
C15B—H15B	0.9500	C25D—H25D	0.9500
C16B—N17B	1.372 (8)	N26D—O27D	1.312 (6)
C16B—H16B	0.9500	O1W—H1W	0.8400
N17B—O18B	1.323 (6)	O1W—H2W	0.8400
C19B—S20B	1.823 (5)	O2W—H2WA	0.8401
C19B—H19C	0.9900	O2W—H2WB	0.8400
C19B—H19D	0.9900	O3W—H3WA	0.8201
S20B—C21B	1.735 (5)	O3W—H3WB	0.8491
C21B—N26B	1.362 (6)	O4W—H4WA	0.8400
C21B—C22B	1.392 (7)	O4W—H4WB	0.8400
C22B—C23B	1.379 (7)	O5W—H5WA	0.8400
C22B—H22B	0.9500	O5W—H5WB	0.8400
C23B—C24B	1.408 (9)	O6W—H6WA	0.8400
C23B—H23B	0.9500	O6W—H6WB	0.8400
C24B—C25B	1.355 (9)	O7W—H7WA	0.8400
C24B—H24B	0.9500	O7W—H7WB	0.8400
C25B—N26B	1.359 (7)	O8W—H8WA	0.8400
C25B—H25B	0.9500	O8W—H8WB	0.8489
N26B—O27B	1.319 (6)	O9W—H9WA	0.8400
C1C—C6C	1.387 (9)	O9W—H9WB	0.8400
C1C—C2C	1.407 (9)	O10W—H10W	0.8400
C1C—C10C	1.510 (9)	O10W—H10X	0.8400
C2C—C3C	1.384 (10)	O11W—H11W	0.8400
C2C—C7C	1.524 (11)	O11W—H11Y	0.8400
C3C—C4C	1.374 (11)	O12W—H12W	0.8400
C3C—H3C	0.9500	O12W—H12X	0.8400
C4C—C5C	1.428 (10)	C1L—O2L	1.26 (2)
C4C—C8C	1.509 (10)	C1L—C3L	1.494 (5)
C5C—C6C	1.407 (9)	C1L—C4L	1.500 (5)
C5C—C19C	1.489 (10)	C3L—H3L1	0.9800
C6C—C9C	1.527 (10)	C3L—H3L2	0.9800
C7C—H7C1	0.9800	C3L—H3L3	0.9800
C7C—H7C2	0.9800	C4L—H4L1	0.9800
C7C—H7C3	0.9800	C4L—H4L2	0.9800
C8C—H8C1	0.9800	C4L—H4L3	0.9800
C8C—H8C2	0.9800		
			
C2A—C1A—C6A	120.3 (4)	C4C—C5C—C19C	121.2 (7)
C2A—C1A—C10A	119.3 (4)	C1C—C6C—C5C	120.7 (6)
C6A—C1A—C10A	120.4 (4)	C1C—C6C—C9C	120.8 (6)
C3A—C2A—C1A	118.4 (4)	C5C—C6C—C9C	118.4 (6)
C3A—C2A—C7A	119.5 (5)	C2C—C7C—H7C1	109.5
C1A—C2A—C7A	122.1 (5)	C2C—C7C—H7C2	109.5
C4A—C3A—C2A	123.3 (5)	H7C1—C7C—H7C2	109.5
C4A—C3A—H3A	118.4	C2C—C7C—H7C3	109.5
C2A—C3A—H3A	118.4	H7C1—C7C—H7C3	109.5
C3A—C4A—C5A	117.7 (5)	H7C2—C7C—H7C3	109.5
C3A—C4A—C8A	120.2 (5)	C4C—C8C—H8C1	109.5
C5A—C4A—C8A	122.1 (5)	C4C—C8C—H8C2	109.5
C6A—C5A—C4A	120.6 (4)	H8C1—C8C—H8C2	109.5
C6A—C5A—C19A	120.2 (4)	C4C—C8C—H8C3	109.5
C4A—C5A—C19A	119.1 (4)	H8C1—C8C—H8C3	109.5
C5A—C6A—C1A	119.6 (4)	H8C2—C8C—H8C3	109.5
C5A—C6A—C9A	120.3 (4)	C6C—C9C—H9C1	109.5
C1A—C6A—C9A	120.1 (4)	C6C—C9C—H9C2	109.5
C2A—C7A—H7A1	109.5	H9C1—C9C—H9C2	109.5
C2A—C7A—H7A2	109.5	C6C—C9C—H9C3	109.5
H7A1—C7A—H7A2	109.5	H9C1—C9C—H9C3	109.5
C2A—C7A—H7A3	109.5	H9C2—C9C—H9C3	109.5
H7A1—C7A—H7A3	109.5	C1C—C10C—S11C	105.9 (4)
H7A2—C7A—H7A3	109.5	C1C—C10C—H10E	110.6
C4A—C8A—H8A1	109.5	S11C—C10C—H10E	110.6
C4A—C8A—H8A2	109.5	C1C—C10C—H10F	110.6
H8A1—C8A—H8A2	109.5	S11C—C10C—H10F	110.6
C4A—C8A—H8A3	109.5	H10E—C10C—H10F	108.7
H8A1—C8A—H8A3	109.5	C12C—S11C—C10C	101.4 (3)
H8A2—C8A—H8A3	109.5	N17C—C12C—C13C	118.0 (5)
C6A—C9A—H9A1	109.5	N17C—C12C—S11C	113.1 (4)
C6A—C9A—H9A2	109.5	C13C—C12C—S11C	128.8 (5)
H9A1—C9A—H9A2	109.5	C14C—C13C—C12C	120.6 (5)
C6A—C9A—H9A3	109.5	C14C—C13C—H13C	119.7
H9A1—C9A—H9A3	109.5	C12C—C13C—H13C	119.7
H9A2—C9A—H9A3	109.5	C15C—C14C—C13C	119.1 (5)
C1A—C10A—S11A	107.5 (3)	C15C—C14C—H14C	120.5
C1A—C10A—H10A	110.2	C13C—C14C—H14C	120.5
S11A—C10A—H10A	110.2	C14C—C15C—C16C	119.9 (6)
C1A—C10A—H10B	110.2	C14C—C15C—H15C	120.0
S11A—C10A—H10B	110.2	C16C—C15C—H15C	120.0
H10A—C10A—H10B	108.5	N17C—C16C—C15C	120.0 (6)
C12A—S11A—C10A	101.1 (2)	N17C—C16C—H16C	120.0
N17A—C12A—C13A	119.5 (5)	C15C—C16C—H16C	120.0
N17A—C12A—S11A	113.1 (4)	O18C—N17C—C16C	120.0 (5)
C13A—C12A—S11A	127.4 (4)	O18C—N17C—C12C	117.7 (5)
C12A—C13A—C14A	119.0 (6)	C16C—N17C—C12C	122.4 (5)
C12A—C13A—H13A	120.5	C5C—C19C—S20C	106.9 (5)
C14A—C13A—H13A	120.5	C5C—C19C—H19E	110.3
C15A—C14A—C13A	119.9 (6)	S20C—C19C—H19E	110.3
C15A—C14A—H14A	120.1	C5C—C19C—H19F	110.3
C13A—C14A—H14A	120.1	S20C—C19C—H19F	110.3
C16A—C15A—C14A	118.9 (6)	H19E—C19C—H19F	108.6
C16A—C15A—H15A	120.6	C21C—S20C—C19C	103.9 (4)
C14A—C15A—H15A	120.6	N26C—C21C—C22C	120.3 (9)
N17A—C16A—C15A	121.9 (6)	N26C—C21C—S20C	109.7 (8)
N17A—C16A—H16A	119.1	C22C—C21C—S20C	129.9 (6)
C15A—C16A—H16A	119.1	C21C—C22C—C23C	121.3 (8)
O18A—N17A—C16A	121.3 (5)	C21C—C22C—H22C	119.4
O18A—N17A—C12A	118.0 (4)	C23C—C22C—H22C	119.4
C16A—N17A—C12A	120.7 (5)	C24C—C23C—C22C	118.4 (11)
C5A—C19A—S20A	106.5 (3)	C24C—C23C—H23C	120.8
C5A—C19A—H19A	110.4	C22C—C23C—H23C	120.8
S20A—C19A—H19A	110.4	C23C—C24C—C25C	121.7 (12)
C5A—C19A—H19B	110.4	C23C—C24C—H24C	119.2
S20A—C19A—H19B	110.4	C25C—C24C—H24C	119.2
H19A—C19A—H19B	108.6	N26C—C25C—C24C	118.9 (11)
C21A—S20A—C19A	101.7 (3)	N26C—C25C—H25C	120.6
N26A—C21A—C22A	119.5 (5)	C24C—C25C—H25C	120.6
N26A—C21A—S20A	112.3 (4)	O27C—N26C—C21C	118.7 (10)
C22A—C21A—S20A	128.2 (4)	O27C—N26C—C25C	122.0 (10)
C23A—C22A—C21A	119.4 (6)	C21C—N26C—C25C	119.3 (11)
C23A—C22A—H22A	120.3	C6D—C1D—C2D	120.5 (5)
C21A—C22A—H22A	120.3	C6D—C1D—C10D	119.7 (6)
C24A—C23A—C22A	119.4 (7)	C2D—C1D—C10D	119.8 (5)
C24A—C23A—H23A	120.3	C3D—C2D—C1D	118.2 (5)
C22A—C23A—H23A	120.3	C3D—C2D—C7D	119.5 (6)
C25A—C24A—C23A	120.7 (6)	C1D—C2D—C7D	122.3 (6)
C25A—C24A—H24A	119.6	C2D—C3D—C4D	122.7 (6)
C23A—C24A—H24A	119.6	C2D—C3D—H3D	118.7
N26A—C25A—C24A	120.3 (6)	C4D—C3D—H3D	118.7
N26A—C25A—H25A	119.8	C3D—C4D—C5D	118.4 (5)
C24A—C25A—H25A	119.8	C3D—C4D—C8D	119.2 (6)
O27A—N26A—C25A	121.3 (5)	C5D—C4D—C8D	122.4 (6)
O27A—N26A—C21A	118.0 (4)	C6D—C5D—C4D	120.1 (5)
C25A—N26A—C21A	120.7 (6)	C6D—C5D—C19D	119.5 (5)
C2B—C1B—C6B	119.5 (4)	C4D—C5D—C19D	120.4 (5)
C2B—C1B—C10B	119.8 (4)	C5D—C6D—C1D	120.0 (6)
C6B—C1B—C10B	120.7 (4)	C5D—C6D—C9D	119.4 (5)
C3B—C2B—C1B	119.1 (4)	C1D—C6D—C9D	120.6 (5)
C3B—C2B—C7B	119.0 (4)	C2D—C7D—H7D1	109.5
C1B—C2B—C7B	121.9 (4)	C2D—C7D—H7D2	109.5
C2B—C3B—C4B	122.1 (4)	H7D1—C7D—H7D2	109.5
C2B—C3B—H3B	118.7	C2D—C7D—H7D3	109.5
C4B—C3B—H3B	118.7	H7D1—C7D—H7D3	109.5
C3B—C4B—C5B	119.0 (4)	H7D2—C7D—H7D3	109.5
C3B—C4B—C8B	119.1 (4)	C4D—C8D—H8D1	109.5
C5B—C4B—C8B	121.9 (4)	C4D—C8D—H8D2	109.5
C4B—C5B—C6B	120.2 (4)	H8D1—C8D—H8D2	109.5
C4B—C5B—C19B	119.6 (4)	C4D—C8D—H8D3	109.5
C6B—C5B—C19B	120.2 (4)	H8D1—C8D—H8D3	109.5
C5B—C6B—C1B	120.1 (4)	H8D2—C8D—H8D3	109.5
C5B—C6B—C9B	120.1 (4)	C6D—C9D—H9D1	109.5
C1B—C6B—C9B	119.8 (4)	C6D—C9D—H9D2	109.5
C2B—C7B—H7B1	109.5	H9D1—C9D—H9D2	109.5
C2B—C7B—H7B2	109.5	C6D—C9D—H9D3	109.5
H7B1—C7B—H7B2	109.5	H9D1—C9D—H9D3	109.5
C2B—C7B—H7B3	109.5	H9D2—C9D—H9D3	109.5
H7B1—C7B—H7B3	109.5	C1D—C10D—S11D	107.8 (4)
H7B2—C7B—H7B3	109.5	C1D—C10D—H10G	110.2
C4B—C8B—H8B1	109.5	S11D—C10D—H10G	110.2
C4B—C8B—H8B2	109.5	C1D—C10D—H10H	110.2
H8B1—C8B—H8B2	109.5	S11D—C10D—H10H	110.2
C4B—C8B—H8B3	109.5	H10G—C10D—H10H	108.5
H8B1—C8B—H8B3	109.5	C12D—S11D—C10D	101.9 (3)
H8B2—C8B—H8B3	109.5	N17D—C12D—C13D	118.6 (7)
C6B—C9B—H9B1	109.5	N17D—C12D—S11D	111.8 (6)
C6B—C9B—H9B2	109.5	C13D—C12D—S11D	129.6 (6)
H9B1—C9B—H9B2	109.5	C12D—C13D—C14D	119.8 (8)
C6B—C9B—H9B3	109.5	C12D—C13D—H13D	120.1
H9B1—C9B—H9B3	109.5	C14D—C13D—H13D	120.1
H9B2—C9B—H9B3	109.5	C15D—C14D—C13D	120.7 (9)
C1B—C10B—S11B	107.1 (3)	C15D—C14D—H14D	119.7
C1B—C10B—H10C	110.3	C13D—C14D—H14D	119.7
S11B—C10B—H10C	110.3	C14D—C15D—C16D	118.9 (8)
C1B—C10B—H10D	110.3	C14D—C15D—H15D	120.6
S11B—C10B—H10D	110.3	C16D—C15D—H15D	120.6
H10C—C10B—H10D	108.6	C15D—C16D—N17D	119.8 (10)
C12B—S11B—C10B	103.3 (2)	C15D—C16D—H16D	120.1
N17B—C12B—C13B	118.9 (5)	N17D—C16D—H16D	120.1
N17B—C12B—S11B	112.1 (4)	O18D—N17D—C12D	117.7 (7)
C13B—C12B—S11B	129.0 (4)	O18D—N17D—C16D	120.1 (8)
C12B—C13B—C14B	120.7 (5)	C12D—N17D—C16D	122.1 (9)
C12B—C13B—H13B	119.6	C5D—C19D—S20D	108.5 (4)
C14B—C13B—H13B	119.6	C5D—C19D—H19G	110.0
C15B—C14B—C13B	119.3 (6)	S20D—C19D—H19G	110.0
C15B—C14B—H14B	120.3	C5D—C19D—H19H	110.0
C13B—C14B—H14B	120.3	S20D—C19D—H19H	110.0
C16B—C15B—C14B	120.1 (6)	H19G—C19D—H19H	108.4
C16B—C15B—H15B	120.0	C21D—S20D—C19D	101.5 (3)
C14B—C15B—H15B	120.0	N26D—C21D—C22D	118.5 (5)
C15B—C16B—N17B	119.8 (6)	N26D—C21D—S20D	112.0 (4)
C15B—C16B—H16B	120.1	C22D—C21D—S20D	129.4 (5)
N17B—C16B—H16B	120.1	C23D—C22D—C21D	120.4 (6)
O18B—N17B—C12B	118.4 (4)	C23D—C22D—H22D	119.8
O18B—N17B—C16B	120.4 (5)	C21D—C22D—H22D	119.8
C12B—N17B—C16B	121.2 (5)	C24D—C23D—C22D	118.7 (6)
C5B—C19B—S20B	106.8 (3)	C24D—C23D—H23D	120.7
C5B—C19B—H19C	110.4	C22D—C23D—H23D	120.7
S20B—C19B—H19C	110.4	C25D—C24D—C23D	121.1 (6)
C5B—C19B—H19D	110.4	C25D—C24D—H24D	119.4
S20B—C19B—H19D	110.4	C23D—C24D—H24D	119.4
H19C—C19B—H19D	108.6	N26D—C25D—C24D	119.4 (6)
C21B—S20B—C19B	101.7 (2)	N26D—C25D—H25D	120.3
N26B—C21B—C22B	119.0 (5)	C24D—C25D—H25D	120.3
N26B—C21B—S20B	112.6 (4)	O27D—N26D—C25D	119.9 (5)
C22B—C21B—S20B	128.3 (4)	O27D—N26D—C21D	118.3 (5)
C23B—C22B—C21B	120.6 (5)	C25D—N26D—C21D	121.8 (5)
C23B—C22B—H22B	119.7	H1W—O1W—H2W	109.5
C21B—C22B—H22B	119.7	H2WA—O2W—H2WB	109.5
C22B—C23B—C24B	118.7 (6)	H3WA—O3W—H3WB	103.1
C22B—C23B—H23B	120.7	H4WA—O4W—H4WB	109.5
C24B—C23B—H23B	120.7	H5WA—O5W—H5WB	109.5
C25B—C24B—C23B	119.4 (6)	H6WA—O6W—H6WB	109.5
C25B—C24B—H24B	120.3	H7WA—O7W—H7WB	109.5
C23B—C24B—H24B	120.3	H8WA—O8W—H8WB	106.7
C24B—C25B—N26B	121.3 (5)	H9WA—O9W—H9WB	109.5
C24B—C25B—H25B	119.3	H10W—O10W—H10X	109.5
N26B—C25B—H25B	119.3	H11W—O11W—H11Y	109.5
O27B—N26B—C25B	120.5 (5)	H12W—O12W—H12X	109.5
O27B—N26B—C21B	118.6 (4)	O2L—C1L—C3L	112.8 (18)
C25B—N26B—C21B	120.9 (5)	O2L—C1L—C4L	116.0 (18)
C6C—C1C—C2C	120.3 (6)	C3L—C1L—C4L	131 (2)
C6C—C1C—C10C	121.6 (6)	C1L—C3L—H3L1	113.5
C2C—C1C—C10C	118.0 (6)	C1L—C3L—H3L2	109.7
C3C—C2C—C1C	118.7 (7)	H3L1—C3L—H3L2	109.5
C3C—C2C—C7C	119.8 (7)	C1L—C3L—H3L3	105.1
C1C—C2C—C7C	121.5 (7)	H3L1—C3L—H3L3	109.5
C4C—C3C—C2C	122.5 (7)	H3L2—C3L—H3L3	109.5
C4C—C3C—H3C	118.7	C1L—C4L—H4L1	107.7
C2C—C3C—H3C	118.7	C1L—C4L—H4L2	110.0
C3C—C4C—C5C	119.2 (7)	H4L1—C4L—H4L2	109.5
C3C—C4C—C8C	119.3 (8)	C1L—C4L—H4L3	110.7
C5C—C4C—C8C	121.4 (8)	H4L1—C4L—H4L3	109.5
C6C—C5C—C4C	118.6 (6)	H4L2—C4L—H4L3	109.5
C6C—C5C—C19C	120.1 (7)		
			
C6A—C1A—C2A—C3A	3.8 (7)	C6C—C1C—C2C—C3C	0.2 (10)
C10A—C1A—C2A—C3A	−177.3 (4)	C10C—C1C—C2C—C3C	−177.4 (7)
C6A—C1A—C2A—C7A	−176.1 (4)	C6C—C1C—C2C—C7C	177.6 (7)
C10A—C1A—C2A—C7A	2.8 (7)	C10C—C1C—C2C—C7C	0.0 (10)
C1A—C2A—C3A—C4A	−2.9 (7)	C1C—C2C—C3C—C4C	−0.5 (12)
C7A—C2A—C3A—C4A	177.0 (5)	C7C—C2C—C3C—C4C	−178.0 (9)
C2A—C3A—C4A—C5A	0.9 (7)	C2C—C3C—C4C—C5C	−0.4 (12)
C2A—C3A—C4A—C8A	−179.3 (5)	C2C—C3C—C4C—C8C	−178.4 (8)
C3A—C4A—C5A—C6A	0.1 (7)	C3C—C4C—C5C—C6C	1.7 (11)
C8A—C4A—C5A—C6A	−179.6 (5)	C8C—C4C—C5C—C6C	179.6 (7)
C3A—C4A—C5A—C19A	−179.0 (4)	C3C—C4C—C5C—C19C	178.6 (7)
C8A—C4A—C5A—C19A	1.3 (7)	C8C—C4C—C5C—C19C	−3.5 (11)
C4A—C5A—C6A—C1A	0.8 (7)	C2C—C1C—C6C—C5C	1.0 (9)
C19A—C5A—C6A—C1A	179.9 (4)	C10C—C1C—C6C—C5C	178.6 (6)
C4A—C5A—C6A—C9A	179.6 (4)	C2C—C1C—C6C—C9C	−176.5 (7)
C19A—C5A—C6A—C9A	−1.3 (7)	C10C—C1C—C6C—C9C	1.1 (9)
C2A—C1A—C6A—C5A	−2.8 (7)	C4C—C5C—C6C—C1C	−2.0 (10)
C10A—C1A—C6A—C5A	178.3 (4)	C19C—C5C—C6C—C1C	−178.9 (6)
C2A—C1A—C6A—C9A	178.4 (4)	C4C—C5C—C6C—C9C	175.6 (7)
C10A—C1A—C6A—C9A	−0.5 (7)	C19C—C5C—C6C—C9C	−1.3 (10)
C2A—C1A—C10A—S11A	−101.7 (4)	C6C—C1C—C10C—S11C	−90.6 (6)
C6A—C1A—C10A—S11A	77.3 (5)	C2C—C1C—C10C—S11C	87.0 (6)
C1A—C10A—S11A—C12A	171.1 (3)	C1C—C10C—S11C—C12C	175.6 (5)
C10A—S11A—C12A—N17A	−171.8 (4)	C10C—S11C—C12C—N17C	175.9 (5)
C10A—S11A—C12A—C13A	7.4 (6)	C10C—S11C—C12C—C13C	−7.2 (6)
N17A—C12A—C13A—C14A	3.2 (9)	N17C—C12C—C13C—C14C	−1.6 (8)
S11A—C12A—C13A—C14A	−176.0 (5)	S11C—C12C—C13C—C14C	−178.3 (5)
C12A—C13A—C14A—C15A	0.7 (10)	C12C—C13C—C14C—C15C	−0.2 (9)
C13A—C14A—C15A—C16A	−3.3 (11)	C13C—C14C—C15C—C16C	0.9 (10)
C14A—C15A—C16A—N17A	2.0 (10)	C14C—C15C—C16C—N17C	0.1 (10)
C15A—C16A—N17A—O18A	179.5 (5)	C15C—C16C—N17C—O18C	176.9 (6)
C15A—C16A—N17A—C12A	1.9 (9)	C15C—C16C—N17C—C12C	−2.0 (10)
C13A—C12A—N17A—O18A	177.8 (5)	C13C—C12C—N17C—O18C	−176.3 (5)
S11A—C12A—N17A—O18A	−2.9 (6)	S11C—C12C—N17C—O18C	0.9 (7)
C13A—C12A—N17A—C16A	−4.6 (8)	C13C—C12C—N17C—C16C	2.7 (9)
S11A—C12A—N17A—C16A	174.7 (4)	S11C—C12C—N17C—C16C	179.9 (5)
C6A—C5A—C19A—S20A	−77.3 (5)	C6C—C5C—C19C—S20C	99.3 (7)
C4A—C5A—C19A—S20A	101.8 (4)	C4C—C5C—C19C—S20C	−77.6 (8)
C5A—C19A—S20A—C21A	−175.6 (3)	C5C—C19C—S20C—C21C	−143.5 (6)
C19A—S20A—C21A—N26A	178.6 (4)	C19C—S20C—C21C—N26C	176.3 (5)
C19A—S20A—C21A—C22A	−2.0 (6)	C19C—S20C—C21C—C22C	−5.2 (8)
N26A—C21A—C22A—C23A	−0.6 (8)	N26C—C21C—C22C—C23C	−1.6 (11)
S20A—C21A—C22A—C23A	−179.9 (5)	S20C—C21C—C22C—C23C	−179.9 (6)
C21A—C22A—C23A—C24A	0.7 (10)	C21C—C22C—C23C—C24C	0.3 (12)
C22A—C23A—C24A—C25A	−0.4 (11)	C22C—C23C—C24C—C25C	2.1 (14)
C23A—C24A—C25A—N26A	0.0 (11)	C23C—C24C—C25C—N26C	−3.0 (15)
C24A—C25A—N26A—O27A	−179.8 (6)	C22C—C21C—N26C—O27C	−179.0 (7)
C24A—C25A—N26A—C21A	0.1 (9)	S20C—C21C—N26C—O27C	−0.4 (9)
C22A—C21A—N26A—O27A	−179.9 (5)	C22C—C21C—N26C—C25C	0.6 (11)
S20A—C21A—N26A—O27A	−0.5 (6)	S20C—C21C—N26C—C25C	179.2 (6)
C22A—C21A—N26A—C25A	0.2 (8)	C24C—C25C—N26C—O27C	−178.8 (9)
S20A—C21A—N26A—C25A	179.7 (4)	C24C—C25C—N26C—C21C	1.6 (13)
C6B—C1B—C2B—C3B	−1.3 (6)	C6D—C1D—C2D—C3D	0.9 (9)
C10B—C1B—C2B—C3B	179.3 (4)	C10D—C1D—C2D—C3D	179.9 (6)
C6B—C1B—C2B—C7B	179.1 (4)	C6D—C1D—C2D—C7D	−177.9 (6)
C10B—C1B—C2B—C7B	−0.3 (7)	C10D—C1D—C2D—C7D	1.1 (9)
C1B—C2B—C3B—C4B	0.0 (7)	C1D—C2D—C3D—C4D	−1.7 (10)
C7B—C2B—C3B—C4B	179.7 (4)	C7D—C2D—C3D—C4D	177.1 (6)
C2B—C3B—C4B—C5B	0.6 (7)	C2D—C3D—C4D—C5D	0.2 (10)
C2B—C3B—C4B—C8B	−179.9 (4)	C2D—C3D—C4D—C8D	−177.7 (7)
C3B—C4B—C5B—C6B	0.1 (7)	C3D—C4D—C5D—C6D	2.1 (9)
C8B—C4B—C5B—C6B	−179.5 (4)	C8D—C4D—C5D—C6D	179.9 (6)
C3B—C4B—C5B—C19B	−178.5 (4)	C3D—C4D—C5D—C19D	−177.2 (6)
C8B—C4B—C5B—C19B	2.0 (7)	C8D—C4D—C5D—C19D	0.6 (9)
C4B—C5B—C6B—C1B	−1.3 (7)	C4D—C5D—C6D—C1D	−2.9 (9)
C19B—C5B—C6B—C1B	177.3 (4)	C19D—C5D—C6D—C1D	176.4 (5)
C4B—C5B—C6B—C9B	177.5 (4)	C4D—C5D—C6D—C9D	176.7 (6)
C19B—C5B—C6B—C9B	−3.9 (7)	C19D—C5D—C6D—C9D	−4.0 (8)
C2B—C1B—C6B—C5B	1.9 (6)	C2D—C1D—C6D—C5D	1.4 (9)
C10B—C1B—C6B—C5B	−178.6 (4)	C10D—C1D—C6D—C5D	−177.7 (5)
C2B—C1B—C6B—C9B	−176.9 (4)	C2D—C1D—C6D—C9D	−178.1 (6)
C10B—C1B—C6B—C9B	2.5 (6)	C10D—C1D—C6D—C9D	2.8 (8)
C2B—C1B—C10B—S11B	96.0 (4)	C6D—C1D—C10D—S11D	−87.3 (6)
C6B—C1B—C10B—S11B	−83.5 (5)	C2D—C1D—C10D—S11D	93.7 (6)
C1B—C10B—S11B—C12B	−176.2 (3)	C1D—C10D—S11D—C12D	163.2 (5)
C10B—S11B—C12B—N17B	177.0 (4)	C10D—S11D—C12D—N17D	179.3 (5)
C10B—S11B—C12B—C13B	−4.0 (6)	C10D—S11D—C12D—C13D	−1.8 (7)
N17B—C12B—C13B—C14B	−0.7 (9)	N17D—C12D—C13D—C14D	1.6 (9)
S11B—C12B—C13B—C14B	−179.6 (5)	S11D—C12D—C13D—C14D	−177.2 (5)
C12B—C13B—C14B—C15B	0.2 (10)	C12D—C13D—C14D—C15D	−2.5 (10)
C13B—C14B—C15B—C16B	0.4 (12)	C13D—C14D—C15D—C16D	2.8 (11)
C14B—C15B—C16B—N17B	−0.4 (13)	C14D—C15D—C16D—N17D	−2.4 (12)
C13B—C12B—N17B—O18B	−178.6 (5)	C13D—C12D—N17D—O18D	−179.8 (7)
S11B—C12B—N17B—O18B	0.6 (7)	S11D—C12D—N17D—O18D	−0.7 (9)
C13B—C12B—N17B—C16B	0.6 (9)	C13D—C12D—N17D—C16D	−1.2 (10)
S11B—C12B—N17B—C16B	179.8 (5)	S11D—C12D—N17D—C16D	177.8 (6)
C15B—C16B—N17B—O18B	179.1 (7)	C15D—C16D—N17D—O18D	−179.9 (8)
C15B—C16B—N17B—C12B	−0.1 (11)	C15D—C16D—N17D—C12D	1.6 (11)
C4B—C5B—C19B—S20B	−97.3 (4)	C6D—C5D—C19D—S20D	88.4 (6)
C6B—C5B—C19B—S20B	84.1 (5)	C4D—C5D—C19D—S20D	−92.3 (6)
C5B—C19B—S20B—C21B	−174.8 (3)	C5D—C19D—S20D—C21D	−159.7 (4)
C19B—S20B—C21B—N26B	176.0 (4)	C19D—S20D—C21D—N26D	−177.4 (4)
C19B—S20B—C21B—C22B	−2.0 (5)	C19D—S20D—C21D—C22D	3.9 (6)
N26B—C21B—C22B—C23B	−1.8 (7)	N26D—C21D—C22D—C23D	−0.6 (8)
S20B—C21B—C22B—C23B	176.1 (4)	S20D—C21D—C22D—C23D	178.1 (4)
C21B—C22B—C23B—C24B	0.6 (8)	C21D—C22D—C23D—C24D	0.7 (8)
C22B—C23B—C24B—C25B	0.8 (9)	C22D—C23D—C24D—C25D	0.6 (8)
C23B—C24B—C25B—N26B	−1.0 (10)	C23D—C24D—C25D—N26D	−2.0 (9)
C24B—C25B—N26B—O27B	−178.9 (6)	C24D—C25D—N26D—O27D	−178.5 (5)
C24B—C25B—N26B—C21B	−0.3 (9)	C24D—C25D—N26D—C21D	2.1 (8)
C22B—C21B—N26B—O27B	−179.7 (5)	C22D—C21D—N26D—O27D	179.8 (5)
S20B—C21B—N26B—O27B	2.1 (6)	S20D—C21D—N26D—O27D	0.8 (6)
C22B—C21B—N26B—C25B	1.7 (8)	C22D—C21D—N26D—C25D	−0.8 (8)
S20B—C21B—N26B—C25B	−176.5 (4)	S20D—C21D—N26D—C25D	−179.7 (4)

### Hydrogen-bond geometry (Å, °)

**Table:**

*D*—H···*A*	*D*—H	H···*A*	*D*···*A*	*D*—H···*A*
O1W—H1W···O18A^i^	0.84	1.98	2.800 (6)	167
O1W—H2W···O3W	0.84	2.13	2.832 (9)	141
O2W—H2WA···O4W	0.84	2.21	2.685 (11)	116
O2W—H2WB···O18C^ii^	0.84	1.87	2.666 (8)	157
O2W—H2WB···N17C^ii^	0.84	2.63	3.324 (8)	141
O3W—H3WB···O4W	0.85	2.00	2.849 (12)	179
O4W—H4WA···O9W	0.84	2.12	2.799 (17)	138
O4W—H4WB···O2W	0.84	2.07	2.685 (11)	129
O5W—H5WA···O27C^iii^	0.84	2.04	2.713 (14)	137
O5W—H5WB···O7W	0.84	2.15	2.817 (17)	137
O6W—H6WB···O5W	0.84	1.95	2.658 (16)	142
O7W—H7WA···O10W^iv^	0.84	2.28	2.74 (2)	115
O7W—H7WB···O5W	0.84	2.31	2.817 (17)	119
O8W—H8WA···O18D	0.84	2.08	2.819 (16)	147
O8W—H8WA···N17D	0.84	2.68	3.339 (17)	136
O8W—H8WB···O27B^v^	0.85	1.96	2.807 (15)	179
O9W—H9WA···O6W	0.84	2.42	3.028 (19)	130
O9W—H9WB···O11W	0.84	2.32	2.84 (3)	120
O10W—H10W···O11W^vi^	0.84	2.33	2.89 (3)	125
O10W—H10X···O7W^i^	0.84	2.10	2.74 (2)	133
O11W—H11Y···O9W	0.84	2.16	2.84 (3)	137
O12W—H12W···O27D	0.84	1.77	2.55 (2)	152
C14B—H14B···Cg1	0.95	2.93	3.548 (7)	124
C23B—H23B···Cg2	0.95	2.94	3.621 (7)	129
C8A—H8A3···Cg3	0.98	2.90	3.796 (6)	152

Symmetry codes: (i) *x*, −*y*+1/2, *z*−1/2; (ii) −*x*+1, −*y*, −*z*+1; (iii) −*x*+2, −*y*, −*z*+1; (iv) *x*, −*y*+1/2, *z*+1/2; (v) *x*+1, −*y*+1/2, *z*+1/2; (vi) −*x*+2, *y*+1/2, −*z*+3/2.
